# Utilizing onion peel extract as photosensitizer combined with 405 nm blue light to control *Salmonella* Typhimurium on eggshells

**DOI:** 10.1111/1750-3841.70167

**Published:** 2025-03-27

**Authors:** Chae‐Yeon Woo, Gi‐Hyeok Lee, Kyung‐Jik Lim, Jun‐Won Kang

**Affiliations:** ^1^ Department of Food Science and Biotechnology Dongguk University‐Seoul Goyang‐si Republic of Korea

**Keywords:** antimicrobial blue light (abl), Onion peel extracts, photosensitizer

## Abstract

**Abstract:**

The use of blue light within a range of 400–470 nm holds significant potential for sanitization purposes. However, due to an extended exposure duration needed for an antibacterial effect, the utilization of a photosensitizer (PS) to increase the efficacy of the treatment becomes essential. This study investigated prospective use of onion peel extract as a PS in combination with 405 nm blue light for the inactivation of *Salmonella* Typhimurium, a common foodborne pathogen on eggs. Extracts were obtained using 99% ethanol, 50% ethanol, and distilled water (DW). Their photosensitizing activities were then compared. The combination of 405 nm blue light and onion peel extract using 99% ethanol reduced bacterial populations more effectively than blue light treatment alone, while also increasing reactive oxygen species generation, cell membrane permeability, lipid peroxidation, and DNA damage levels. However, the antimicrobial effect of the 99% ethanol extract did not show a concentration dependence. Spraying DW extract on eggshell treated with 99% ethanol onion peel extract at 1 mg/mL and blue light further enhanced *Salmonella* reduction. Liquid chromatography was conducted for component separation. However, none of the separated fractions exhibited a significant antibacterial effect, suggesting that the active compounds responsible for antibacterial activity might work synergistically in the crude extract rather than individually. In contrast, the crude extract exhibited a significant antibacterial effect, suggesting that 99% ethanol‐extracted onion peel can serve as a PS, particularly in its crude state without purification, and effectively inactivate *Salmonella* on eggshells.

**Practical Application:**

Antimicrobial blue light (aBL) in the range of 400–470 nm is a promising nonthermal technology with several advantages, including minimal impact on food quality and safety. This study optimized the concentration of onion peel extract to improve its effectiveness as a photosensitizer in aBL treatment against *Salmonella* Typhimurium on eggshells. These results may serve as a reference for further optimizing aBL treatments, offering a potentially sustainable and cost‐effective photosensitizer for pathogen control.

## 1 INTRODUCTION

Eggs are an inexpensive source of animal protein for humans, with the lowest environmental impact. Eggs contain nearly 40 kinds of proteins and provide 18 different amino acids, nine of which are essential amino acids. They also contain vitamins (A, D, E, B1, B2, and B3) and minerals (Fe, Zn, Cu, Mg, and I) (Luksienė & Zukauskas, 2009; Molnár & Szőllősi, 2020). Global egg production has increased from 80.4 million metric tons in 2018 to 87 million metric tons in 2020 (Guyonnet, 2023). Unfortunately, a positive correlation exists between increased egg consumption and the incidence of salmonellosis attributed to *Salmonella* Typhimurium and *Salmonella* Enteritidis with symptoms including stomach cramps, diarrhea, and vomiting (M. J. Cardoso et al., 2021).

There are two primary pathways through which *Salmonella* contamination can occur in eggs. The first pathway involves pre‐egg formation contamination, wherein a direct transmission of *Salmonella* occurs within the yolk, yolk membranes, and albumen. This contamination route occurs when hens orally ingest *Salmonella*, leading to bacterial infiltration inside the egg before forming the eggshell or egg membrane. The second pathway of *Salmonella* contamination in eggs is through eggshell contamination, which can occur due to vaginal infections or fecal contamination. Particularly, following oviposition, the presence of manure and moist organic material in the hatchery or nesting environment provides a conducive environment for *Salmonella* growth (Gantois et al., 2009). *Salmonella* on the surface of eggshell has the ability to penetrate both the eggshell and its membrane, leading to internal contamination of the egg (Messens et al., 2005). Furthermore, eggshell contamination by *Salmonella* presents a significant concern as it can lead to cross‐contamination, increasing the risk of transmitting the bacteria to other surfaces, utensils, and/or food products, potentially causing foodborne illness outbreaks (Pande et al., 2016). Therefore, proper hygiene practices and thorough cleaning and disinfection of surfaces are essential for mitigating various issues that can arise from eggshell contamination, including the risk of direct consumption of contaminated egg or cross‐contamination to other surfaces, utensils, and food products.

Although pasteurization methods such as hot air, hot water, and infrared radiation have demonstrated efficacies in inactivating *Salmonella* on eggshells, these treatments carry the risk of egg cracking. They can also negatively impact the nutritional quality and functional properties of eggs (Lasagabaster et al., 2011). Thermal treatments typically induce protein denaturation and gel network formation in the egg albumen, leading to altered rheological properties and undesirable textural characteristics in both egg white and yolk proteins, ultimately resulting in decreased consumer acceptability (Kiosseoglou & Paraskevopoulou, 2005). Currently, chlorine (sodium hypochlorite) is the predominant chemical employed by the majority of egg producers for egg washing purposes owing to its ability to efficiently eliminate a wide range of pathogenic organisms through the oxidation of cellular materials (Wan et al., 2017). Nevertheless, chlorine can undergo reactions with natural organic substances, leading to the creation of disinfection byproducts that may possess carcinogenic properties (Lasagabaster et al., 2011). Additionally, the use of washing chemicals can cause detrimental effects on the protective cuticle layer of eggs, facilitating increased microbial infiltration into egg contents (Munoz et al., 2015). Therefore, to address limitations of traditional methods, the utilization of more innovative non‐thermal techniques is crucial for providing effective pathogen inactivation on eggshells and preserving their quality attributes.

The use of light‐emitting diode (LED) technology holds significant potential for sanitization purposes owing to its cost‐effectiveness, durability, and versatility in various applications across the food supply chain (D'Souza et al., 2015). Ultraviolet C (UVC) radiation in the range of 200–280 nm, which can induce the formation of pyrimidine dimers in the DNA of pathogens, has been proven to be able to control *Salmonella* contamination on eggshells (Bing et al., 2019; Holck et al., 2018; Keklik et al., 2010). However, the practical application of UVC in the food industry is limited due to its low penetration ability for solids or opaque liquids as well as its potential risks of causing severe eye and skin damage to food operators and sensory degradation of food products (M.‐J. Kim et al., 2016). To address these challenges, antimicrobial blue light (aBL) in the spectrum of 405–470 nm presents a promising alternative to UVC. Blue light in this wavelength range has been shown to exhibit higher antimicrobial efficacy than other visible light spectra (Guffey & Wilborn, 2006; Guyonnet, 2023). aBL has also been reported to exhibit strong antimicrobial activity against both Gram‐negative and Gram‐positive bacteria (Chen et al., 2023; Larkin, 2022; Murdoch et al., 2012). It can penetrate through plastics, glass, water, and fabrics (Maclean et al., 2014; McKenzie et al., 2013). One notable benefit of 405 nm LEDs is their ability to provide continuous, long‐duration (e.g., hours to days) background disinfection, effectively reducing microbial contamination in food processing and medical settings. Since this wavelength is not harmful to humans, allowing for continuous use without posing risk to humans or disrupting daily activities (Chen et al., 2023). The mechanism of action of aBL is still under investigation. Although it is not fully identified yet, the most widely accepted hypothesis suggests that aBL can stimulate endogenous photosensitizing chromophores such as iron‐free porphyrins and flavins within cells, leading to the generation of cytotoxic reactive oxygen species (ROS) (Dai et al., 2012; Hamblin et al., 2005). However, a prolonged irradiation time is required, ranging from hours to days, for the bactericidal effect of 405 nm or 460 nm light. This presents a significant disadvantage. To mitigate this drawback and reduce treatment time, the utilization of a photosensitizer (PS) becomes essential in the process (D.‐K. Kim et al., 2022).

Onions are the most commonly cultivated crop in the world. The total global production of onion is 93.23 million tons worldwide each year (Kumar et al., 2022). However, non‐edible parts of onions, including the top, root, onion skin, and peels, contribute to a significant amount of waste, with European countries producing an annual onion waste of 0.6 million tons (Katsampa et al., 2015). California generates 100,000 tons of onion waste per year (French et al., 2010). Even domestic waste in Brazil's food stalls ranges from 0.04 to 0.26 kg per day (Brancoli et al., 2022). Furthermore, onion waste cannot be reused as a fertilizer due to its high sulfur content. If not properly disposed of, it can have detrimental effects on the environment (Benítez et al., 2011; Sagar et al., 2022). The onion peels harbor a substantial amount of bioactive compounds, including quercetin, polyphenols, flavonoids, and flavanols (Kumar et al., 2022). Notably, quercetin and polyphenols have been extensively evaluated as suitable substances for utilization as PSs in aBL applications (I.‐H. Lee et al., 2023; Wang et al., 2017). These findings strongly suggest that onion peel extract could serve as a valuable PS for aBL applications; however, this potential remains largely unexplored. While several studies have demonstrated the antimicrobial effects of onion peel extracts against *Salmonella* strains, they all required relatively high concentrations exceeding 10 mg/mL to achieve inhibitory effects (Joković et al., 2024; Mihajilov‐Krstev et al., 2022; Sagar & Pareek, 2020). For example, Joković et al. (2024) reported that concentrations as high as 25 mg/mL were necessary to achieve antimicrobial effects against *Salmonella* Enteritidis. The use of such high concentrations poses challenges for food applications from a sensory perspective, limiting its practical use as an antimicrobial agent. Therefore, further research is needed to explore the potential of PSs capable of effectively controlling pathogens at lower concentrations.

To the authors’ knowledge, no data are available on the photosensitizing properties of onion peel extracts against *S*. Typhimurium on eggshell. Therefore, the aim of this study was twofold: (i) To investigate how different extraction solvents (distilled water [DW], 50% ethanol, 99% ethanol) and extract concentrations (0.1, 0.5, and 1 mg/mL) affect the yield of phenolic compounds and their antibacterial activity, optimizing onion peel extracts for PS applications. The inactivation mechanisms against *S*. Typhimurium were evaluated through measurements of intracellular ROS generation, cell membrane permeability, lipid peroxidation (LPO), and DNA damage. (ii) To assess the combined antimicrobial effects of multi‐component polyphenol mixtures, the onion peel extract was fractionated into six distinct fractions, and the effects of each individual fraction were compared to those of the crude extract. Additionally, quercetin content was quantitatively analyzed to determine its specific contribution to the antimicrobial activity against *S*. Typhimurium.

## 2 MATERIALS AND METHODS

### 2.1 Onion peel extraction preparation

Onion peel was dried for two days in a dry oven and crushed with a mixer to form powder. Powdered onion peel was soaked in sterile DW, 50% ethanol, or 99% ethanol solution (1:10 w/v) at 60°C for 20 min. Extraction solution was filtered through a paper filter and concentrated using a rotary evaporator to reduce solvent content. The concentrate was fivefold diluted with DW and freeze‐dried. Onion peel extract stock solution was prepared by dissolving the freeze‐dried powder in 99% ethanol.

### 2.2 Preparation of bacterial strain and inoculum

Three strains of *S*. Typhimurium (DT104, ATCC 43,971, ATCC 19,585) were obtained from the Bacterial Culture Collection at Seoul National University (Seoul, Korea). To create stock cultures, these strains were cultured in 5 mL of tryptic soy broth (TSB, MB cell, Kisanbio), mixed with 50% glycerol at a ratio of 7:3 (v/v), and preserved at −80°C. To prepare working cultures, stock solution of each strain was streaked onto tryptic soy agar (TSA, MB cell, Kisanbio) plates and incubated at 37°C for 24 h. Subsequently, these cultures were stored at 4°C for future use up to 7 days.

Cultivation of each *S*. Typhimurium strain was carried out in 5 mL of TSB at 37°C for 24 h. These cultured strains were combined and centrifuged at 4000 g for 20 min at 4°C and washed three times with sterile phosphate buffer saline (PBS, pH 7.4). Resultant pellets were reconstituted with 10 mL of PBS, resulting in an approximate bacterial density of 10^8^ to 10^9^ CFU/mL.

### 2.3 Experimental apparatus and treatment

An LED chip (D.‐K. Kim et al., 2022) emitting 405 nm wavelength was connected to an electronic printed circuit board. Aluminum heat sinks were affixed to the backside of LEDs to prevent excessive heat buildup. A constant electric current of 1.024 mA was applied through a DC power supply (TPM series; Toyotech, Incheon). The distance between the LED module and a Petri dish (60 mm × 15 mm) was established at 5 cm.

The treatment solution was prepared by dissolving 0.5 mL of onion peel extract stock solution in 4.5 mL of PBS, placed in a 60 mm × 15 mm Petri dish. The onion peel extract stock solutions were initially prepared at concentrations of 1, 5, and 10 mg/mL by dissolving the freeze‐dried extract, obtained through extraction with 99% ethanol, 50% ethanol, and DW, in ethanol. These solutions were further diluted 10‐fold in PBS during treatment to achieve final concentrations of 0.1, 0.5, and 1.0 mg/mL, respectively, and used as treatment solutions. For the control group, to which only light treatment was applied, 0.5 mL of 99% ethanol was added instead of the stock solution to maintain the same composition as treatment group. Treatment samples were treated at a dose up to 191.8 J/cm^2^ at room temperature (21  ±  1°C) with or without LED module while being mixed with a magnetic stirrer at 250 rpm.

A spectrometer (AvaSpec‐ULS2048CL‐EVO‐UA‐50; Avantes) was employed to measure the intensity of the LED module to calculate treatment dose (J/cm^2^) by multiplying the irradiance value by the irradiation time. The light intensity in this study treatment set was calculated as 36.11 mW/cm^2^.

### 2.4 Bacterial enumeration

Treated samples were subjected to 10‐fold serial dilution in 9 mL of sterile 0.2% buffered peptone water (PW; MB cell, Kisanbio), followed by plating 0.1 mL aliquots of either samples or dilutions onto selective media, Xylose lysine desoxycholate agar (XLD, MB cell, Kisanbio). After incubation at 37°C for 24 h, typical colonies (black colonies) were enumerated and expressed as log_10_ CFU/mL.

### 2.5 Mathematical modeling

Survival curves of *S*. Typhimurium were fitted with the Weibull model using GInaFit, a freely available software tool utilized for evaluating microbial survivor patterns (Geeraerd et al., 2005). Parameters (*δ* and *p*) of the Weibull model were determined by employing the following equation:

$$\log\left(N\right)=\;\log\left(N_{0}\right)-{\left(\frac{t}{\delta }\right)}^{p}$$

where *N* (CFU/mL) was the population of pathogens, *N*_0_ was the initial population, *t* (sec) was the treatment time, *δ* (sec) was the time for the first decimal reduction, and *p* was the parameter related to shape of the line. The *D*_3d_ or *D*_5d_ value, which was the dose required to reduce *S*. Typhimurium by 3 or 5 log, was calculated using the above equation and derived parameters.

### 2.6 Comparison of photocatalytic activity between fractions of onion peel extract

Amberlite XAD‐4 resin (Sigma‐Aldrich) was used to obtain fractions of onion peel extraction. In preparation for the experiment, the resin underwent an activation process, which included multiple washes using ethanol and DW to ensure optimal cleanliness and readiness. After 20 mL of onion peel extraction solution (10 mg/mL) was loaded onto the top of the dried resin (50 g) packed in a glass column (inner diameter: 5 cm; height: 30 cm), 500 mL of 70% ethanol (mobile phase) was then loaded onto the top of the resin. While allowing the mobile phase to descend to the bottom by gravity, six fractions were sequentially obtained. Obtained fractions were concentrated using a rotary evaporator and then freeze‐dried. As described in the procedure, a surviving population of *S*. Typhimurium was derived by applying the LED module to the obtained fraction at a concentration of 0.5 mg/mL.

### 2.7 Procedures of treating eggshells

Eggs were purchased from a local grocery store (Goyang‐si, South Korea). The inside contents of each eggs were taken out. Eggshells were washed using 70% ethanol to remove any impurities that might be on these shells. Afterward, shells were dried for 24 h at room temperature in laminar flow hood. Dried eggshells cut into 1.5 cm by 1.5 cm pieces. A 25 µL aliquot of the previously described bacterial inoculum (8 log CFU/mL) was spot inoculated onto each piece. These inoculated eggshell pieces were dried for 1 h at room temperature and used for experiments.

Treatment solutions were prepared by dissolving stock solution with DW at a 1:4 ratio (v:v). Each onion peel extract solution adjusted to concentrations of 0, 0.5, and 1 mg/mL was sprayed once onto an inoculated eggshell (one piece) using a sprayer. The amount sprayed once with the sprayer was 100 µL. These sprayed eggshells were immediately exposed to blue light at a dose of 191.8 J/cm^2^ using the same experimental apparatus as described above. When the irradiation dose reached 95.9 J/cm^2^, DW was sprayed once onto the eggshell using a sprayer to enhance the reduction of bacteria.

Treated samples were transferred into sterile stomacher bags containing 25 mL of 0.2% PW. Stomacher bags were homogenized with a stomacher for 1 min. After homogenization, the homogenized sample was 10‐fold serially diluted in 9 mL of sterile 0.2% PW. Then 0.1 mL of appropriate dilution was spread‐plated onto XLD agar. These plates were incubated at 37°C for 24 h. Typical colonies were enumerated and expressed as log_10_ CFU/sample.

To enumerate injured cells of S. Typhimurium, the overlay method was used. One‐tenth‐milliliter aliquots of appropriate dilutions were spread plated onto TSA (a nonselective medium) to facilitate repair of injured cells, and the plates were incubated at 37°C for 2 h to enable injured cells to recover. The plates were then overlaid with 7 mL of the selective medium XLD. After the overlay was solidified, plates were incubated at 37°C for an additional 22 h, and then typical black colonies were counted.

### 2.8 Measurements of cell membrane damage, total intracellular ROS, and DNA damage

Experiments were conducted to determine the inactivation mechanism. Note 1 mL of inoculum (8 log CFU/mL) was inoculated into each treatment solution and treated with 405 nm blue light at a dose of 191.8 J/cm^2^ under the same conditions as described above to investigate cell membrane damage, total intracellular ROS, and DNA damage.

Fluorescent dye propidium iodine (PI; Sigma‐Aldrich), diphenyl‐1‐pyrenylphosphine (DPPP; aladdin), and CM‐$H_{2}$ DCFDA [5‐(and‐6)‐chloromethyl‐2′,7′‐dichlorodihydrofluorescein diacetate; Invitrogen] were used to determine the degree of cell membrane damage, LPO, and intracellular ROS, respectively. Treated samples were washed twice with PBS, and final pellets were resuspended with PI, DPPP, or CM‐$H_{2}$ DCFDA solution at a final concentration of 2.9, 50 µM, or 5 µM and incubated for 10, 20, or 15 min at 37°C, respectively. After incubation, cells were collected by centrifugation at 13,500 g for 10 min and washed twice with PBS to remove unreacted reagent. Final pellets were resuspended with PBS. Fluorescence was measured with a spectrofluorophotometer (F‐7100; Hitachi) at excitation/emission wavelengths of 493/630 nm for PI uptake assay, 351/380 nm for DPPP assay, or 495/520 nm for intracellular ROS assay.

SYBR Green I [2‐[N‐(3‐dimethylaminopropyl)‐Npropylamino]‐4‐[2,3‐dihydro‐3‐methyl‐(benzo‐1,3‐thiazol‐2‐yl) methylidene]‐1‐phenylquinolinium; Invitrogen was used to assess DNA damage (Han et al., 2016). After treatment, treated cells were washed twice with PBS, and intracellular DNA was released by sonication for 2 min (1 s on and 1 s off) using an ultrasonic homogenizer (UH‐700Z; UMC Science) in an ice bath. Cells were centrifuged at 10,000 × *g* for 10 min to obtain supernatant. SYBR Green I (1:10,000 dilution; Molecular Probes) was applied to the supernatant at a working concentration (1:1). Cells were incubated at 37°C for 15 min. After incubation, fluorescence was measured with a spectrofluorophotometer at excitation and emission wavelengths of 485 nm and 525 nm, respectively.

### 2.9 LC‐MS/MS analysis

To quantify quercetin content in onion peel extract, high‐performance liquid chromatography tandem mass spectrometry (HPLC‐MS/MS) was performed. For sample preparation, onion peel extracts obtained using three different solvents (99% ethanol, 50% ethanol, and DW) and quercetin were dissolved in methanol (Honeywell) at a concentration of 1 mg/mL and filtered through 0.22 nm membrane filters. Then 4 µL of each filtrate was injected into an LC‐MS/MS system (API 3200; Sciex). Separation was performed on a C18 column from Suspelco (10 cm × 4.6 mm, 5 µm of particle size) at a flow rate of 0.3 mL/min and an oven temperature of 40°C. A linear solvent gradient of binary mobile phase (solvent A: 0.5% formic acid in DW; solvent B: 0.5% formic acid in acetonitrile) was applied as follows: 98% A/2% B at 0–1 min, 2% A/98% B at 1.5–4.2 min, and 98% A/2% B at 5.2 min.

Quercetin (TGI) and caffeine (Daesung) were both analyzed in a positive‐ionization mode and a multiple reaction monitoring mode (MRM) with the following optimized MS condition: (a) ion spray voltage and interface temperature of 4500 V and 550°C, respectively; (b) nebulizer gas and auxiliary gas pressure of 55 psi; (c) curtain gas pressure of 21 psi; (d) declustering potential of 67 and 55 V for quercetin and caffeine, respectively; (e) entrance potential of 6 V and 5 V; (f) collision energy at 10 V; and (g) collision exit potential at 10 V. Ion transitions of Q1:Q3 303.1/153.3 m/z for quercetin and Q1:Q3 195.2/138 m/z for caffeine (internal standard) were used for quantification. Linearity, limit of detection (LOD), and limit of quantification (LOQ) were calculated. Analyst 1.3 software (Sciex) was used for data acquisition and processing.

### 2.10 Total phenolic content

Total phenolic contents in onion peel extracts were determined using the Folin–Ciocalteu method (Singleton et al., 1999). Briefly, the onion peel extract was prepared at a concentration of 1 mg/mL by dissolving it in ethanol. A 400 µL aliquot of the extract was mixed with 100 µL of Folin–Ciocalteu reagent (Junsei). After incubating for 5 min, 500 µL of a sodium carbonate solution (4 g/100 mL; Daejung) was added. Following incubation at room temperature for 1 h, the absorbance of the clear solution was measured at 765 nm. To construct the calibration curve, various concentrations of a gallic acid (Daejung) solution were mixed with the same reagent as described above. The total phenolic content was expressed as gallic acid equivalent in milligrams per gram of onion peel extract.

### 2.11 Statistical analysis

All experiments were conducted in triplicate. Data are expressed as means of values ± standard deviations. They were analyzed using Statistical Analysis System (SAS) Version 9.4 (SAS Institute Inc.). All statistical analyses were performed using analysis of variance and Kruskal–Wallis test. Bonferroni test was performed as a post hoc when Kruskal–Wallis test results were statistically significant. Significant differences between values were considered when *p* was <0.05.

## 3 RESULTS AND DISCUSSION

### 3.1 Antimicrobial effect of onion peel extract with 405 nm blue light against *S*. Typhimurium

Viable counts of *S*. Typhimurium after treatment with 405 nm blue light and onion peel extract prepared with 99% ethanol, 50% ethanol, or DW (0%) at different concentrations (0.1, 0.5, and 1.0 mg/mL) were investigated (Table 1). From a dose of 38.36 J/cm^2^ onward, 99% ethanol extract at a concentration of 0.5 mg/mL exhibited additional antimicrobial effects compared to 405 nm blue light treatment alone (*p *< 0.05). At concentrations of 0.1 mg/mL and 1 mg/mL for the 99% ethanol extract, antimicrobial effects were significantly (*p* < 0.05) increased starting from blue light dose of 115.08 and 153.44 J/cm^2^ compared to 405 nm blue light only treatment. After blue light treatment at a dose of 191.8 J/cm^2^, the 99% ethanol extract at 0.1 mg/mL, 0.5 mg/mL, and 1 mg/mL concentrations showed log reductions of 4.22, 5.57, and 4.68, respectively, with the 0.5 mg/mL concentration consistently exhibiting the most pronounced efficacy throughout the treatment period. Furthermore, the antimicrobial effect of onion peel extracts prepared with 0%, 50%, and 99% EtOH, respectively, was evaluated at a concentration of 1 mg/mL without 405 nm blue light treatment. As a result, no reduction in the *S*. Typhimurium population was observed until the treatment time reached 1 h 40 min, corresponding to the maximum treatment dose (191.8 J/cm^2^) in this study (data not shown). However, it was known that onion peel extracts are known for their antimicrobial properties due to their high phenolic content, while the specific component responsible for the antimicrobial activity is still unclear (Joković et al., 2024; W. J. Kim et al., 2011; K. A. Lee et al., 2011). This result is likely due to the significantly lower concentration of extracts used in our study compared to previous research, which typically applied concentrations above 10 mg/mL to achieve antimicrobial effects against *Salmonella* strains (Joković et al., 2024; Mihajilov‐Krstev et al., 2022; Sagar & Pareek, 2020). Therefore, the antimicrobial effect observed under blue light exposure can be attributed to photocatalytic activity only.

**TABLE 1:** Log reductions of *Salmonella* Typhimurium after treatment with onion peel extract and 405 nm blue light.

Treatment solution	Concentration (mg/mL)	Treatment dose (J/cm^2^)					
		0 (0 min)	38.36 (20 min)	76.72 (40 min)	115.08 (100 min)	153.44 (120 min)	191.8 (140 min)
		Log reduction [log_10_(*N*_0_/*N*)]					
Control	–	0.00 ± 0.00A	0.76 ± 0.01A	1.75 ± 0.02A	2.31 ± 0.02AB	2.71 ± 0.06AD	2.97 ± 0.09AB
Extraction from onion peel with 0% ethanol (DW)	0.1	0.00 ± 0.00A	0.79 ± 0.04AB	1.29 ± 0.02B	1.97 ± 0.06B	2.44 ± 0.03A	2.81 ± 0.07A
	0.5	0.00 ± 0.00A	0.98 ± 0.10BCE	1.78 ± 0.05A	2.19 ± 0.06AB	2.90 ± 0.07BCD	3.28 ± 0.16BC
	1	0.00 ± 0.00A	1.04 ± 0.10CDE	1.65 ± 0.05A	2.05 ± 0.20AB	2.82 ± 0.19BD	3.23 ± 0.05ABC
Extraction from onion peel with 50% ethanol	0.1	0.00 ± 0.00A	0.90 ± 0.05ABE	1.82 ± 0.02BC	2.19 ± 0.11AB	3.11 ± 0.13BC	3.61 ± 0.05C
	0.5	0.00 ± 0.00A	0.74 ± 0.09A	1.70 ± 0.08A	2.18 ± 0.15AB	3.18 ± 0.16C	3.53 ± 0.20C
	1	0.00 ± 0.00A	0.94 ± 0.03ABCE	1.21 ± 0.15B	2.26 ± 0.14AB	2.73 ± 0.12AD	3.40 ± 0.03BC
Extraction from onion peel with 99% ethanol	0.1	0.00 ± 0.00A	1.12 ± 0.07CD	1.64 ± 0.07A	2.74 ± 0.16CD	4.04 ± 0.04E	4.22 ± 0.11D
	0.5	0.00 ± 0.00A	1.21 ± 0.01D	2.01 ± 0.07C	3.04 ± 0.10C	3.67 ± 0.06F	5.57 ± 0.15E
	1	0.00 ± 0.00A	1.02 ± 0.08CDE	1.77 ± 0.04B	2.41 ± 0.01AD	3.59 ± 0.14F	4.78 ± 0.32F

*Note*: Values are presented as means ± standard deviations from three replications. Means with different uppercase letters within the same column indicate significant differences (*p* < 0.05). *N*_0_, initial population; N, population after treatment.

Abbreviation: DW, distilled water.

For the 50% ethanol extract, concentrations of 0.1 mg/mL and 0.5 mg/mL displayed heightened antimicrobial effects compared to 405 nm blue light only treatment starting from a dose of 153.44 J/cm^2^ (*p* < 0.05). At a dose of 191.8 J/cm^2^, there were no significant differences in antimicrobial effects among concentrations of 50% ethanol extract used (*p* > 0.05). Concentrations of 0.1 mg/mL, 0.5 mg/mL, and 1 mg/mL of the 50% ethanol extract showed log reductions of 3.61, 3.53, and 3.40, respectively. DW extract exhibited increased antimicrobial effects than the 405 nm blue light only treatment (*p* < 0.05) from a dose of 191.8 J/cm^2^ onward, with log reductions of 2.81, 3.28, and 3.23 for concentrations of 0.1, 0.5, and 1 mg/mL, respectively. Therefore, the antimicrobial effect of the onion peel extract combined with 405 nm blue light was more effective when 99% ethanol was used as an extraction solvent compared to the use of 50% ethanol or DW (0%) as the extraction solvent. It was plausible that 50% ethanol and DW did not effectively extract photosensitizing compounds from onion peel. Different concentrations of ethanol might also exhibit varying efficiencies in extracting specific compounds.

Table 2 illustrates total phenolic contents of onion peel extracts. Onion peels are enriched with polyphenols, making these byproducts advantageous when they are utilized as functional materials (Kumar et al., 2022). These polyphenols can be used as PSs. The 99% ethanol extract had the highest total phenolic content (765.9 mg of gallic acid/g of extract). It was significantly (*p* < 0.05) higher than the 50% ethanol or DW extract. Phenolic contents of 50% ethanol and DW extracts were 591.06 and 525.46 mg of gallic acid/g of extract, respectively. Therefore, due to its higher polyphenol content acting as a PS, the 99% ethanol extract may offer the most effective antimicrobial result. The polarity of a solvent could lead to variations in the types and amounts of bioactive compounds extracted from onion peels (Bozinou et al., 2023).

**TABLE 2:** Total phenolic and quercetin contents in onion peel extracts.

Extraction solvents	Total phenolic content (mg of gallic acid/g of extract)	Quercetin content (mg of quercetin/g of extract)	Linearity (*R* ^2^)	LOD (ppb)	LOQ (ppb)
DW	525.46 ± 29.34A	23.39 ± 0.51A	0.9999	2.54	7.69
50% Ethanol	591.06 ± 8.25B	46.28 ± 3.87AB	0.9972	10.28	31.14
99% Ethanol	765.90 ± 4.36C	67.71 ± 17.71B	0.9989	2.58	7.82

*Note*: Values are presented as means ± standard deviations from three replications. Means with different uppercase letters within the same column indicate significant differences (*p* < 0.05). Linearity (*R* ^2^), limit of detection (LOD), and limit of quantification (LOQ) were measured for quercetin quantification.

Abbreviation: DW, distilled water.

**FIGURE 1:**
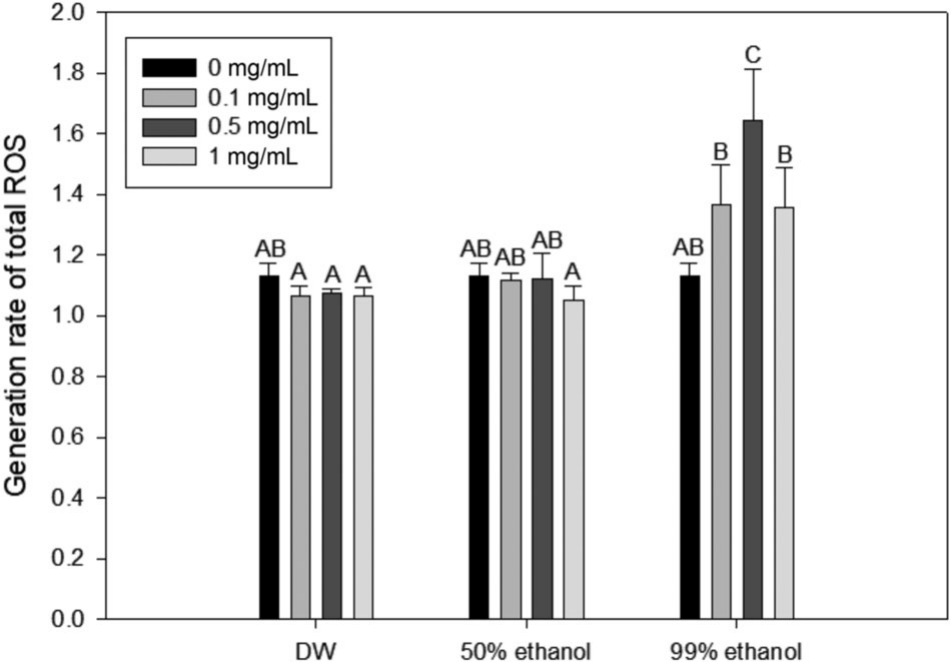
Intracellular reactive oxygen species (ROS) generation values of *Salmonella* Typhimurium after treatment with onion peel extracts and 405 nm blue light (191.8 J/cm^2^). Data are presented as average values of three independent experiments. Standard deviations are shown by error bars. Different letters within the same extraction solvent cluster of treatments indicate significant differences (*p* < 0.05). Data were normalized by dividing fluorescence values obtained from untreated cells using the following formula: fluorescent value after treatment/fluorescent value of untreated control. Distilled water (DW), 50% ethanol, and 99% ethanol refer to solvents used for onion peel extraction.

Interestingly, in our study, antimicrobial effect of the 99% ethanol extract did not improve with higher concentrations. The highest antimicrobial effect was observed at a concentration of 0.5 mg/mL. Nakamura et al. (2015) have investigated antimicrobial activities of polyphenols (caffeic acid, gallic acid, chlorogenic acid, epigallocatechin, epigallocatechin gallate, and proanthocyanidin) exposed to 400 nm blue light against *Staphylococcus aureus*, *Streptococcus mutans*, *Escherichia coli*, and *Pseudomonas aeruginosa*. Their findings revealed that these polyphenols significantly enhanced the inactivation efficiency against all bacteria when exposed to 400 nm blue light alone. However, some phenolics could also inhibit photosensitization‐induced biological damages. The phenolic compound present in Piper betel, namely, allyl pyrocatechol, has been found to effectively protect photosensitization‐mediated LPO in rat liver mitochondria by quenching singlet oxygen (Mula et al., 2008). Huvaere and Skibsted (2015) have confirmed that some flavonoids, such as riboflavin and chlorophyll, can deactivate PS by inhibiting triplet‐excited states of PSs. Moreover, D. R. Cardoso et al. (2006) have illustrated the necessity of employing substantial amounts of phenolic compounds (>0.3 M) to hinder triplet‐excited riboflavin that generates photosensitized ROS. These studies suggest that phenolic compounds not only serve as PSs but also possess inhibitory effects on photosensitization. The concentration of phenolic compounds also seems to play a crucial role in inhibiting photosensitization. Therefore, the 99% ethanol extract, which contained a substantial amount of phenolic compounds, particularly at a concentration of 1 mg/mL, might yield phenolic compounds that could prevent photosensitization‐induced biological damages by reducing the generation of ROS. This could potentially explain the reduced log reduction of the 99% ethanol extract at 1 mg/mL against *S*. Typhimurium compared to that at 0.5 mg/mL, as some extracted phenolic compounds present in mg/mL 99% ethanol extract might impede the action of PSs. This aligned with the reduction in ROS generation observed for the 1 mg/mL of the 99% ethanol extract than the 0.5 mg/mL of the 99% ethanol extract (Figure 1). This result emphasizes the importance of selecting appropriate concentrations and extraction methods when utilizing food byproducts as PSs.

### 3.2 Mathematical modeling

Modeling of survival curves was conducted to quantitatively compare inactivation effects of different onion peel extracts. Survival curves were fitted to a suitable model equation to derive $D_{3}d$ and $D_{5}d$ values, which were dosages required to achieve 3‐log and 5‐log reductions, respectively. Upon evaluation of various models, we observed that the Weibull model provided an excellent fit to the inactivation data, as indicated by its high $R^{2}$ values (≥0.98). This suggested that the model could effectively capture the trend of pathogen inactivation, providing strong evidence for its suitability in this context. Parameters of the model, $D_{3}d$ and $D_{5}d$ values, are shown in Table 3. This result revealed that when cells were treated with the 99% onion peel extract and 405 nm blue light simultaneously, $D_{3}d$ and $D_{5}d$ values were significantly (*p* < 0.05) decreased compared to those for cells treated with 405 nm blue light alone.

**TABLE 3:** *D*_3d_ and *D*_5d_ values using the Weibull model equation of *Salmonella* Typhimurium after treatment with onion peel extraction and 405 nm blue light.

Treatment solution	Concentration (mg/mL)	Weibull				
		*δ* (mean ± SE)	*p* (mean ± SE)	*R* ^2^	$D_{3d}$ (mean ± SE)	$D_{5d}$ (mean ± SE)
DW	–	37.27 ± 1.56	0.70 ± 0.02	0.98	181.06 ± 6.92A	377.72 ± 19.17A
Extraction from onion peel with 0% ethanol (DW)	0.1	51.36 ± 1.62	0.80 ± 0.01	0.99	202.82 ± 4.67B	384.15 ± 8.91A
	0.5	19.08 ± 0.97	0.74 ± 0.02	0.99	162.42 ± 3.65AB	324.88 ± 8.37BC
	1	21.81 ± 2.20	0.76 ± 0.02	0.99	175.74 ± 10.09AC	342.67 ± 13.04B
Extraction from onion peel with 50% ethanol	0.1	41.14 ± 2.63	0.84 ± 0.02	0.99	152.52 ± 4.91C	280.56 ± 5.18D
	0.5	23.23 ± 0.75	0.89 ± 0.02	0.99	153.32 ± 9.62C	272.42 ± 15.91D
	1	27.46 ± 3.56	0.94 ± 0.08	0.99	169.65 ± 5.97AC	292.84 ± 3.59DC
Extraction from onion peel with 99% ethanol	0.1	38.10 ± 2.66	0.92 ± 0.03	0.98	125.04 ± 4.19D	217.34 ± 4.55E
	0.5	23.25 ± 1.43	1.12 ± 0.05	0.98	118.62 ± 3.17D	187.02 ± 3.43E
	1	26.78 ± 1.26	1.15 ± 0.08	0.99	133.48 ± 2.83D	208.33 ± 10.96E

*Note*: *D*_3d_ and *D*_5d_ values are dosages necessary for achieving 3 and 5‐log reductions, respectively. SE, standard error, *R* ^2^, regression coefficient. Values are presented as means ± standard deviations from three replications. Mean values with different uppercase letters within the same column are significantly different (*p* < 0.05).

Abbreviation: DW, distilled water.

### 3.3 Application of onion peel extract with 405 nm blue light for inactivating *S*. Typhimurium on eggshells

**FIGURE 2:**
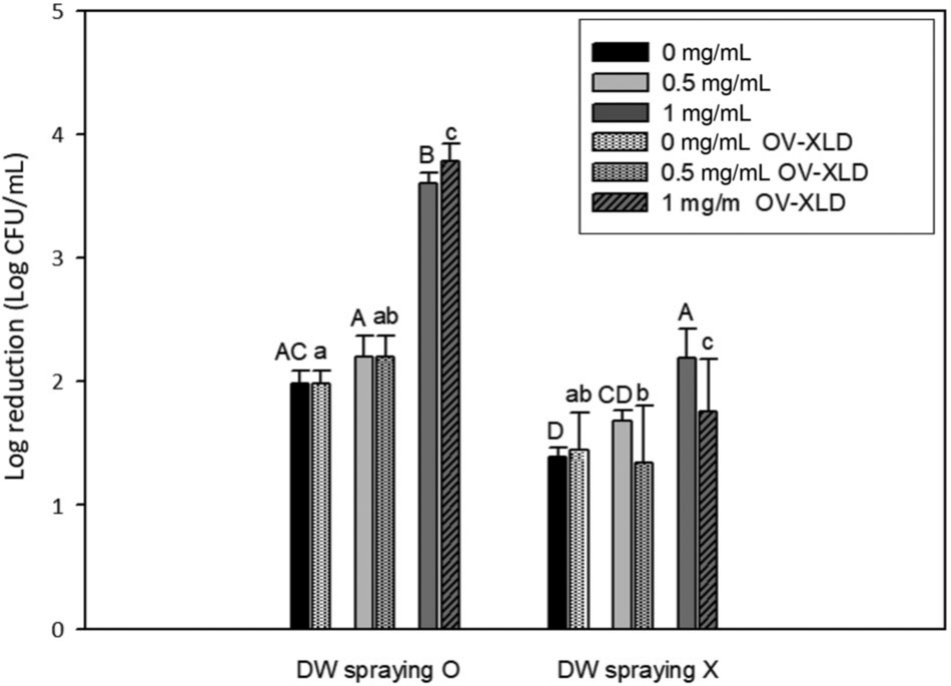
Log reduction of *Salmonella* Typhimurium on eggshells after treatment with both onion peel extract with 99% ethanol and 405 nm blue light (191.8 J/cm^2^) along with distilled water (DW) spraying. Data are presented as average values of three independent experiments. Standard deviations are shown by error bars. Different letters within the same method cluster of treatments indicate significant differences (*p* < 0.05). Xylose lysine desoxycholate (XLD), xylose lysine deoxycholate agar; OV‐XLD, overlay XLD agar on tryptic soy agar (TSA); log reduction (log_10_ [*N*_0_/*N*)]), *N*_0_, initial population; *N*, population after treatment.

Figure 2 illustrates log reductions of *S*. Typhimurium on eggshells following various treatments. Eggshells treated with both 1 mg/mL of the 99% ethanol extract and 405 nm blue light along with intermittent DW spraying in between exhibited the highest reduction rate (*p* < 0.05). The XLD overlay recovery test also confirmed that the bacteria were not injured but rather killed, as no recovery was observed (*p* < 0.05). Unlike our previous results, where the 0.5 mg/mL 99% onion peel extract had the most potent antimicrobial effect in PBS solution, the 1 mg/mL 99% onion peel extract was found to be the most effective on eggshells. Certain substances present in eggshells might have interacted with the onion peel extract, reducing its photosensitizing ability, thus necessitating a higher concentration to exert its antimicrobial effect. Additionally, DW spraying might have diluted the onion peel extract, thereby lowering its concentration to a level similar to 0.5 mg/mL.

Furthermore, results of DW spraying revealed higher (*p* < 0.05) reduction across all concentrations in comparison with treatment without DW spraying. During exposure to 405 nm blue light, the application of DW spray to eggshells could prevent excessive drying of the egg surface. In a study conducted by Bartusik et al. (2012), singlet oxygen bubbles were utilized to neutralize *E. coli* and *Aspergillus fumigatus*. In this process, singlet oxygen diffused from the gas bubble into a water‐based solution and effectively interacted with targeted microorganisms. As a result, when DW was sprayed, ROS could be more effectively transferred into the aqueous media, leading to an increased antibacterial effect. This result shows the potential of combining an onion peel extract with 405 nm blue light supplemented by intermittent DW spraying as an effective approach for reducing *Salmonella* on eggshells. These findings also highlight the importance of maintaining sufficient moisture during photosensitization of onion peel extract for optimal antibacterial results.

### 3.4 Photocatalytic inactivation mechanism

Several studies have suggested that the mechanism of bacterial inactivation utilizing visible light wavelengths requires three elements: light within visible wavelength range, PS, and oxygen (Dai et al., 2012; M.‐J. Kim et al., 2015; Luksienė & Zukauskas, 2009). Upon exposure to light, PS can absorb light energy and undergo excitation. These PSs can transit from a low ground energy state to an excited singlet state and subsequently to an excited triplet state. This process leads to the generation of ROS. During the transition process, the PS can interact with the external environment in two ways. First, an electron can transfer from the substrate to the PS, forming unstable ions that can produce ROS by reacting again with the substrate. Resulting products include superoxide anions, hydroxyl radicals, and hydrogen peroxide. Meanwhile, through energy transfer from triplet‐state PSs to ground‐state molecular oxygen, singlet oxygen radicals are formed. Generated ROS can induce cytotoxic effects by interacting with DNA, RNA, proteins, and lipids, ultimately resulting in the demise of bacterial cells (Cossu et al., 2021; Kim & Yuk, 2017; Luksienė & Zukauskas, 2009).

Thus, the effectiveness of an onion peel extract as a PS could be assessed by measuring intracellular ROS generation values using a CM‐H_2_DCFD probe. CM‐H_2_DCFD can enter cells easily and hydrolyze to produce carboxylate anion dichlorofluorescein inside cells. When ROS oxidize it, it transforms into an extremely fluorescent 2′,7′‐dichlorofluorescein (DCF). A higher fluorescence value of DCF indicates a higher extent of intracellular ROS generation (Kang & Kang, 2019). As shown in Figure 1, ROS generation value of simultaneous treatment with the 405 nm blue light and 99% ethanol extract was higher than individual 405 nm blue light treatment across all concentrations, with 0.5 mg/mL of the 99% ethanol extract resulting in significantly (*p* < 0.05) higher ROS value than blue light treatment alone. However, ROS generation values of 405 nm blue light treatment with 50% ethanol and DW extract were not significantly different (*p* > 0.05) from blue light treatment alone at any concentrations tested. Microorganisms possess internal photosensitizing agents, including porphyrins, flavins, cytochromes, and NADH, which can be activated by exposure to blue light (Hessling et al., 2017). These endogenous PSs of bacteria may lead to ROS generation and cell death with 405 nm blue light treatment alone. However, it was confirmed that 99% ethanol onion peel extract could induce more ROS generation. Thus, it can be utilized as a green PS.

**FIGURE 3:**
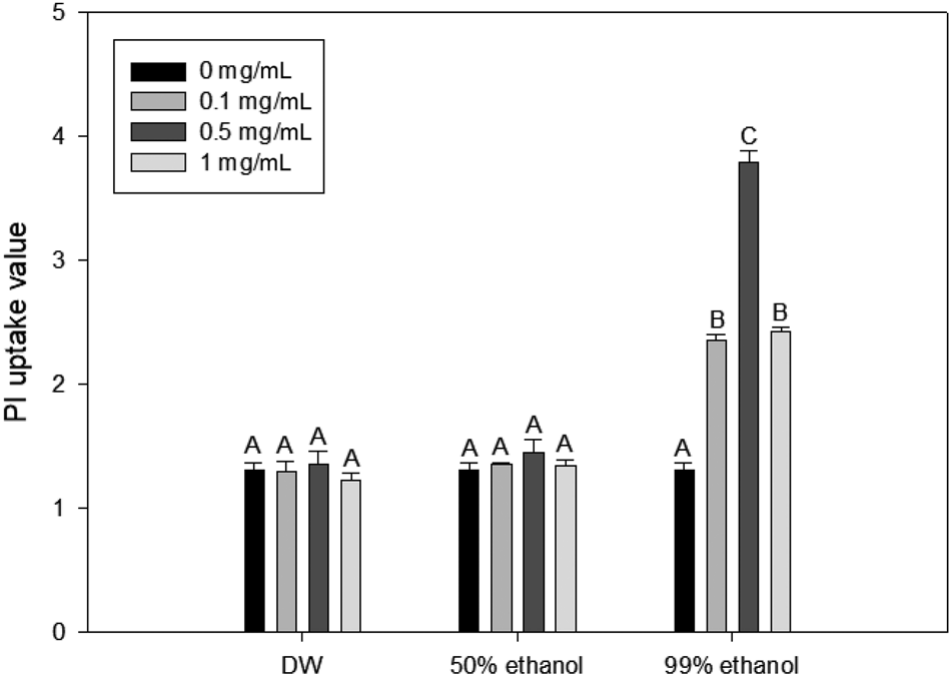
Propidium iodine (PI) uptake values for cell membranes of *Salmonella* Typhimurium after treatment with onion peel extract and 405 nm blue light (191.8 J/cm^2^). Values were obtained from PI uptake assays. Data are presented as average values of three independent experiments. Standard deviations are shown by error bars. Different letters within the same extraction solvent cluster of treatments indicate significant differences (*p* < 0.05). Data were normalized by dividing fluorescence values obtained from untreated cells using the following formula: fluorescent value after treatment/fluorescent value of untreated control. Distilled water (DW), 50% ethanol, and 99% ethanol refer to solvents used for onion peel extraction.

**FIGURE 4:**
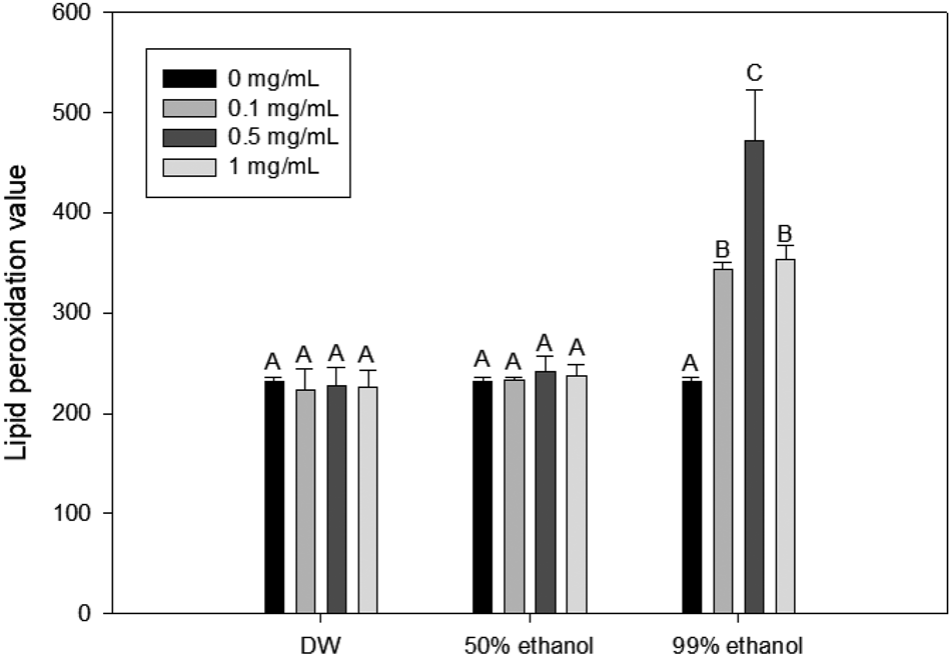
Lipid peroxidation values for cell membranes of *Salmonella* Typhimurium after treatment with onion peel extract and 405 nm blue light (191.8 J/cm^2^). Values were obtained from diphenyl‐1‐pyrenylphosphine (DPPP) uptake assays. Data are presented as average values of three independent experiments. Standard deviations are shown by error bars. Different letters within the same extraction solvent cluster of treatments indicate significant differences (*p* < 0.05). Data were normalized by dividing fluorescence values obtained from untreated cells using the following formula: fluorescent value after treatment/fluorescent value of untreated control. Distilled water (DW), 50% ethanol, and 99% ethanol refer to solvents used for onion peel extraction.

ROS are generally known to cause cell membrane damage, a principal instigator of LPO in cell membranes, ultimately leading to cell death by enhancing membrane permeability (Joshi et al., 2011; Lovrić et al., 2005; Von Moos & Slaveykova, 2014; Wickens, 2001). Therefore, PI uptake assay was conducted to determine membrane permeability. PI can penetrate damaged cell membranes but not intact cells. After entering the cell, PI can interact with nucleic acids to emit enhanced fluorescence. Therefore, the rise in PI uptake value indicates an elevation in cell membrane permeability, signifying the presence of physical damage, such as the formation of pores in the cell membrane (Pagán & Mackey, 2000). PI uptake value during concurrent treatment with 405 nm blue light and a 99% ethanol extract at all concentrations tested was significantly (*p* < 0.05) higher than that during individual blue light treatment (Figure 3). Conversely, the PI uptake value for blue light treatment with 50% ethanol or DW extract did not show a significant difference (*p *> 0.05) from that for 405 nm blue light treatment alone. Therefore, it can be inferred that simultaneous treatment with 99% onion peel extract and 405 nm blue light can result in damage of cell membrane, which might hinder cells from maintaining homeostasis, ultimately resulting in cell death (Park & Kang, 2013). Moreover, it is important to identify which specific component within the cell membrane undergoes alterations that ultimately result in damage through pore formation, leading to enhanced permeability to clarify the cell death mechanism. Fatty acids are essential components in cellular structures. They are mainly found in the bacterial cell membrane as acyl constituents of phospholipids. LPO induced by ROS in the cell membrane can cause cellular damage, leading to reduced fluidity, diminished cell membrane potential, increased permeability, and eventual cell death (Gutteridge, 1995; Kaneda, 1991). Figure 4 shows the degree of LPO of bacterial cell membrane using DPPP assay. LPO value during concurrent treatment with 405 nm blue light and 99% ethanol at all concentrations tested was significantly (*p* < 0.05) higher than that during individual blue light treatment. Conversely, the LPO value for the 405 nm blue light treatment along with 50% ethanol or DW extract did not show a significant (*p* > 0.05) difference from the blue light treatment alone.

**FIGURE 5:**
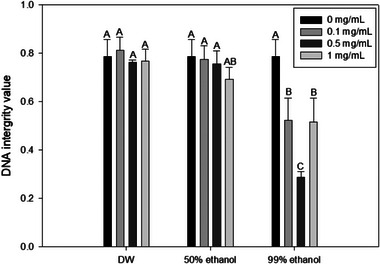
DNA integrity values of *Salmonella* Typhimurium after treatment with onion peel extract with 405 nm blue light (191.8 J/cm^2^). Data are presented as average values of three independent experiments. Standard deviations are shown by error bars. Different letters within the same extraction solvent cluster of treatments indicate significant differences (*p* < 0.05). Data were normalized by dividing fluorescence values obtained from untreated cells using the following formula: fluorescent value after treatment/fluorescent value of untreated control. distilled water (DW), 50% ethanol, and 99% ethanol refer to solvents used for onion peel extraction.

It is believed that cellular DNA is the primary focal point of ROS produced through LED light exposure (M.‐J. Kim et al., 2016). Therefore, DNA damage upon treatment with 405 nm blue light with or without onion peel extract was measured using SYBR Green I. SYBR Green I exhibits a remarkable capacity to significantly enhance brightness by >1000‐fold upon binding with double‐stranded DNA (dsDNA), making it a valuable tool for quantifying nucleic acids (Dragan et al., 2012). Consequently, it serves as an indicator of DNA damage, as any impairment to DNA integrity could result in a reduction of the fluorescence signal emitted by SYBR Green I. Results of DNA integrity are shown in Figure 5. Simultaneous treatment with blue light and a 99% ethanol extract at all concentrations tested showed significantly (*p* < 0.05) lower DNA integrity values than treatment with 405 nm blue light alone. In contrast, the DNA integrity value for 405 nm blue light treatment with 50% ethanol or DW extract did not exhibit a significant difference (*p *> 0.05) compared to that of 405 nm blue light treatment alone. Several studies have indicated that DNA damage is caused by ROS generated from visible light, in accordance with our results. Kim and Yuk (2017) have analyzed the oxidized derivative of 8‐hydroxy‐2′‐deoxyguanosine (8‐OHdG) to investigate whether LED illumination can induce DNA oxidation. Specifically, 8‐hydroxy‐2′‐deoxyguanosine (8‐OHdG) is a biomarker of oxidative DNA damage, formed by the oxidation of guanine in DNA. It is commonly used to assess the extent of oxidative stress within cells. Their findings indicated a significant increase in the level of 8‐OHdG in *S*. Enteritidis cells upon LED illumination, providing confirmation that exposure to a 405 ± 5 nm LED could lead to oxidation of genomic DNA by generating intracellular ROS. Rapacka‐Zdonczyk et al. (2019) have demonstrated that the use of sublethal blue light on *S*. aureus can induce DNA damage, leading to activation of the RecA gene and SOS response. This in turn can enhance the release of error‐prone DNA polymerases, which are enzymes accountable for an increase in mutation rate. Liang et al. (2015) have confirmed that photochemical reactions triggered by blue light with flavin mononucleotide could lead to inactivation of *E. coli* through the production of ROS, causing damage to nucleic acids.

Therefore, increased membrane permeability and loss of DNA integrity were confirmed to be the causes of cell death induced by treatment with both onion peel extract and 405 nm blue light. Several studies have found that the generated ROS can induce oxidative damage not only to lipids but also to various constituents such as proteins in a multitude of other microorganisms (Gogniat & Dukan, 2007; Gogniat et al., 2006; Kang & Kang, 2019; Maness et al., 1999). Consequently, our results represent only partial aspects of the cell demise mechanism. Further research is imperative for a comprehensive understanding.

### 3.5 Component analysis

Onion peel contains over 77 times higher concentrations of flavonoids, particularly polyphenol quercetin, than edible part of onions (Kang et al., 1998; S.‐W. Kim et al., 2019). I.‐H. Lee et al. (2023) have determined antimicrobial activities using quercetin and 405 nm blue light against *E. coli* and confirmed that quercetin can be used as a PS. Therefore, it is hypothesized that the observed photodynamic inactivation of *Salmonella* may be primarily due to the action of quercetin.

LC‐MS/MS quantification was performed to analyze quercetin contents in 99% ethanol, 50% ethanol, and DW onion peel extracts. Table 2 shows quercetin contents of these onion peel extracts. The quercetin content in the 99% ethanol extract was the highest (67.71 mg/1 g of extract). It was significantly (*p *< 0.05) higher than that in the extract obtained with DW (23.39 mg/g extract). The quercetin content in the 50% ethanol extract was 46.28 mg/g extract. The linearity (*R* ^2^) was greater than 0.99 for all samples. LODs for 99% ethanol, 50% ethanol, and DW extracts were 2.58, 10.24, and 2.54 ppb, respectively. Limits of quantification (LOQs) for 99% ethanol, 50% ethanol, and DW extracts were 7.82, 31.14, and 7.69 ppb, respectively. These results revealed that the quercetin contents in the 99% and 50% ethanol extracts were not significantly different (*p* > 0.05) despite their differences in antimicrobial effects. This meant that quercetin did not act as the primary PS responsible for the antimicrobial effect observed in this study.

The combined action of multiple polyphenols, rather than a single one, appears to have contributed to the enhanced antibacterial effect. This has been demonstrated in several studies where combinations of polyphenolic compounds show synergistic antibacterial effects. For instance, the combination of an olive mill wastewater fraction enriched with natural phenolic ingredients hydroxytyrosol, verbascoside, and tyrosol, along with gallic acid resulted in a significant reduction in the minimal inhibitory concentration against Gram‐positive bacteria (*Streptococcus pyogenes* and *Staphylococcus aureus*) and Gram‐negative bacteria (*Escherichia coli* and *Klebsiella pneumoniae*) (Tafesh et al., 2011). Similarly, synergistic antibacterial effect has been found for a combination of theaflavin and epicatechin against all isolates of *Acinetobacter baumannii* and *Stenotrophomonas maltophilia* (Betts et al., 2011). Additionally, Bozinou et al. (2023) have suggested that specific combination of compounds present in a 75% ethanol onion peel extract might lead to enhanced antibacterial activity compared to those present in extracts with other solvents. Therefore, it could be hypothesized that this synergistic effect could also occur when combined with 405 nm blue light.

To investigate this hypothesis, low‐pressure liquid chromatography was conducted to fractionate the 99% ethanol extract into six fractions. This process aimed to isolate specific component acting as a PS that might be responsible for the inactivation of *Salmonella*. Table 4 shows reduction levels by the crude extract and its six fractions. Specifically, the log reduction for the crude extract was 2.89 CFU/mL, while log reductions for fractions 1, 2, 3, 4, 5, and 6 were 1.53, 1.34, 1.43, 2.55, 2.33, and 1.90 CFU/mL, respectively. Despite variations in photocatalytic activity resulting from differences in extracted components through fractionation, no fraction surpassed (*p* < 0.05) the photocatalytic activity of the crude extract. As a result, it can be interpreted that the antimicrobial effect manifests when components are combined. Although the photosensitizing activity of onion peel extract might have derived from various substances, utilizing it in its crude form was proved to be more effective than isolating and utilizing individual compounds. Therefore, applying onion peel extract in its original form can enhance its antibacterial effectiveness. Additionally, the tentative identification of phenolic compounds in 99% ethanol onion peel extracts focused on those with potential antimicrobial activity as PS (Table S1). Among the sixteen identified compounds, ferulic acid, coumaric acid, benzoic acid, and their derivatives have been reported in previous studies to exhibit bactericidal effects against Gram‐negative bacteria under Ultraviolet A radiation (UV‐A) irradiation by promoting oxidative radical generation (de Oliveira et al., 2021; Shirai, Kajiura, Omasa, et al., 2015; Shirai, Kajiura, Matsumura, et al., 2015). Gallic acid also exhibited antimicrobial activity by acting as a PS (PS) against Gram‐negative bacteria under 400 nm LED light (Nakamura et al., 2015). Additionally, natural flavonoid glycosides, particularly kaempferol, show promise as PSs for antimicrobial photodynamic therapy targeting Human Papillomavirus‐associated oropharyngeal cancer (Pourhajibagher & Bahador, 2024). Therefore, these identified phenolic acids may further enhance antimicrobial effect against *S*. Typhimurium through combined effect. Utilizing onion peel extract as a PS has an advantage in that it does not require a purification process of specific substances while still attaining maximum antibacterial effects. This highlights the practical and economical benefit of using its crude extract, as it can maximize antibacterial efficacy while reducing costs and time associated with purification processes.

**TABLE 4:** Reduction of *Salmonella* Typhimurium after treatment with 405‐nm blue light (118 J/cm^2^) and onion peel extract (crude) obtained with 99% ethanol and its six fractions (designated as fractions 1–6).

Fraction	Log reduction
Crude	2.89 ± 0.10A
1	1.53 ± 0.14BD
2	1.35 ± 0.10B
3	1.43 ± 0.12B
4	2.56 ± 0.18C
5	2.33 ± 0.13C
6	1.90 ± 0.14D

*Note*: Data are presented as average values of three independent experiments. Standard deviations are shown by error bars. Log reduction (log_10_[*N*_0_/*N*]), N_0_, initial population; N, population after treatment. Mean values with different uppercase letters within the same column are significantly different (*p* < 0.05).

## 4 CONCLUSIONS

This study investigated the potential of using onion peel extract as a PS in combination with 405 nm blue light to inactivate *S*. Typhimurium. Findings indicated that combining 99% ethanol onion peel extract with 405 nm blue light could significantly diminish bacterial populations, suggesting the potential of onion peel extract derived from 99% ethanol as a promising resource for PS. Component analysis revealed that onion peel extract could be utilized as a PS in its crude form without the need for a specific component purification, offering economic and practical advantages. Furthermore, onion peel extract treatment on eggshell holds significant promise for enhancing food safety by efficiently addressing *Salmonella* contamination on eggshells, thereby contributing to the prevention of foodborne illnesses. Additionally, the utilization of discarded onion peel extract as a PS can play a role in managing food byproducts. This study suggests that onion peel extract has potential for use in various forms as a food safety technology.

## AUTHOR CONTRIBUTIONS

**Chae‐Yeon Woo**: Investigation; formal analysis; methodology; visualization; writing—original draft. **Gi‐Hyeok Lee**: Writing—original draft; methodology; investigation; formal analysis. **Kyung‐Jik Lim**: Methodology; resources; investigation. **Jun‐Won Kang**: Writing—review and editing; conceptualization; supervision; project administration.

## CONFLICT OF INTEREST STATEMENT

The authors declare no conflicts of interest.

## Supporting information



Supporting Information
