# Supplementary material for: Utilizing onion peel extract as photosensitizer combined with 405 nm blue light to control Salmonella Typhimurium on eggshells
Source: J Food Sci. 2025 Mar 27;90(3):e70167. doi: 10.1111/1750-3841.70167 (PMC11948953; doi:10.1111/1750-3841.70167)
Supplement: Supplementary file 1 — Supporting Information [file JFDS-90-0-s001.docx]

***Supplementary material***

**Utilizing onion peel extract as photosensitizer combined with 405 nm blue light to control *Salmonella* Typhimurium on eggshells**

Chae-Yeon Woo^1^, Gi-Hyeok Lee^1^, Kyung-Jik Lim and Jun-Won Kang ^*^

*Department of Food Science and Biotechnology, Dongguk University-Seoul, 32, Dongguk-ro, Ilsandong-gu, Goyang-si, Gyeonggi-do 10326, Republic of Korea*

*Correspondence: Jun-Won Kang, Department of Food Science and Biotechnology, Dongguk University-Seoul, Gyeonggi-do, Republic of Korea, E-mail: junwon89@dgu.edu

^1^These authors contributed equally to this work.

**Table S1.** Tentative identification of onion peel extract from 99% ethanol in negative ionization mode.

| **No** | **Tentative identification** | **Chemical**  **formula** | **Exact molecular mass (*m/z*)** | **Precursor ion**  ***(m/z)*** | **Product ion**  ***(m/z)*** | **Reference** |
| --- | --- | --- | --- | --- | --- | --- |
| 1 | p-hydroxybenzoic acid | C_7_H_6_O_3_ | 138.12 | 137.7 | 93.1 | (Zhong et al., 2020) |
| 2 | Protocatechuic acid | C_7_H_6_O_4_ | 154.12 | 153.2 | 109.2, 108.2 | (Kumar et al., 2015) |
| 3 | Gallic acid | C_7_H_6_O_5_ | 170.12 | 169.2 | 125.3, 107.2 | (Kumar et al., 2015) |
| 4 | Ferulic acid | C_10_H_10_O_4_ | 194.18 | 193.2 | 149, 134.1 | (Sinosaki et al., 2020) |
| 5 | Lunularin | C_14_H_14_O_2_ | 214.26 | 213.4 | 107.2, 106.2 | (Pallauf et al., 2019) |
| 6 | Fisetin | C_15_H_10_O_6_ | 286.24 | 285.3 | 157.2, 145 | (McNab et al., 2009) |
| 7 | Luteolin | C_15_H_10_O_6_ | 286.24 | 285.3 | 241.4, 217.3 | (Gates & Lopes, 2012) |
| 8 | Kaempferol | C_15_H_10_O_6_ | 286.24 | 285.3 | 151 | (Gates & Lopes, 2012) |
| 9 | Dihydrokaempferol | C_15_H_12_O_6_ | 288.25 | 287.5 | 259.2, 243.1 | (Lech, 2020) |
| 10 | Quercetin | C_15_H_10_O_7_ | 302.23 | 301.3 | 179.1, 151.4 | (Dadge et al., 2023) |
| 11 | Taxifolin | C_15_H_12_O_7_ | 304.25 | 303.1 | 285 | (Yang et al., 2016) |
| 12 | Myricetin | C_15_H_10_O_8_ | 318.23 | 317.2 | 179.3, 151 | (Lin et al., 2012) |
| 13 | 3-p-coumaroylquinic acid | C_16_H_18_O_8_ | 338.31 | 337.4 | 191, 163 | (Ali et al., 2024) |

**References**

Ali, A., Mueed, A., Cottrell, J. J., & Dunshea, F. R. (2024). LC-ESI-QTOF-MS/MS Identification and Characterization of Phenolic Compounds from Leaves of Australian Myrtles and Their Antioxidant Activities. *Molecules*, *29*(10), 2259.

Chandradevan, M., Simoh, S., Mediani, A., Ismail, N. H., Ismail, I. S., & Abas, F. (2020). UHPLC‐ESI‐Orbitrap‐MS Analysis of Biologically Active Extracts from Gynura procumbens (Lour.) Merr. and Cleome gynandra L. Leaves. *Evidence‐Based Complementary and Alternative Medicine*, *2020*(1), 3238561.

Dadge, S. D., Syed, A. A., Husain, A., Valicherla, G. R., & Gayen, J. R. (2023). Simultaneous Estimation of Quercetin and trans-Resveratrol in Cissus quadrangularis Extract in Rat Serum Using Validated LC-MS/MS Method: Application to Pharmacokinetic and Stability Studies. *Molecules*, *28*(12), 4656.

Gates, P. J., & Lopes, N. P. (2012). Characterisation of flavonoid aglycones by negative ion chip‐based nanospray tandem mass spectrometry. *International journal of analytical chemistry*, *2012*(1), 259217.

Kumar, S., Chandra, P., Bajpai, V., Singh, A., Srivastava, M., Mishra, D., & Kumar, B. (2015). Rapid qualitative and quantitative analysis of bioactive compounds from Phyllanthus amarus using LC/MS/MS techniques. *Industrial Crops and Products*, *69*, 143-152.

Lech, K. (2020). Universal analytical method for characterization of yellow and related natural dyes in liturgical vestments from Krakow. *Journal of Cultural Heritage*, *46*, 108-118.

Lin, Y., Wu, B., Li, Z., Hong, T., Chen, M., Tan, Y., Jiang, J., & Huang, C. (2012). Metabolite identification of myricetin in rats using HPLC coupled with ESI-MS. *Chromatographia*, *75*, 655-660.

McNab, H., Ferreira, E. S., Hulme, A. N., & Quye, A. (2009). Negative ion ESI–MS analysis of natural yellow dye flavonoids—An isotopic labelling study. *International Journal of Mass Spectrometry*, *284*(1-3), 57-65.

Pallauf, K., Chin, D., Günther, I., Birringer, M., Lüersen, K., Schultheiß, G., Vieten, S., Krauß, J., Bracher, F., & Danylec, N. (2019). Resveratrol, lunularin and dihydroresveratrol do not act as caloric restriction mimetics when administered intraperitoneally in mice. *Scientific Reports*, *9*(1), 4445.

Sinosaki, N., Tonin, A. P., Ribeiro, M. A., Poliseli, C. B., Roberto, S. B., Silveira, R. d., Visentainer, J. V., Santos, O. O., & Meurer, E. C. (2020). Structural study of phenolic acids by triple quadrupole mass spectrometry with electrospray ionization in negative mode and H/D isotopic exchange. *Journal of the Brazilian Chemical Society*, *31*, 402-408.

Yang, C.-J., Wang, Z.-B., Mi, Y.-Y., Gao, M.-J., Lv, J.-N., Meng, Y.-H., Yang, B.-Y., & Kuang, H.-X. (2016). UHPLC-MS/MS determination, pharmacokinetic, and bioavailability study of taxifolin in rat plasma after oral administration of its nanodispersion. *Molecules*, *21*(4), 494.

Zhong, B., Robinson, N. A., Warner, R. D., Barrow, C. J., Dunshea, F. R., & Suleria, H. A. (2020). LC-ESI-QTOF-MS/MS characterization of seaweed phenolics and their antioxidant potential. *Marine drugs*, *18*(6), 331.
